# Engendering Governance After Armed Conflict: Observations From Kosovo

**DOI:** 10.3389/fsoc.2021.574225

**Published:** 2021-05-17

**Authors:** Brigitte M. Holzner

**Affiliations:** Herbert C. Kelman Institute for Interactive Conflict Transformation (HKI), Vienna, Austria

**Keywords:** gender governance, equality, post-conflict, state-building, security, CEDAW, UNSCR

## Abstract

This article addresses gender-responsive governance reforms in post-conflict Kosovo from two perspectives: (1) the perspective of human rights as fundamental for state-building, and (2) the resolutions of the UN Security Council regarding Women, Peace, and Security (WPS), notably the initial UNSCR 1325. Special focus is laid on women's agency in governance matters—at the level of the state and at the level of civil society. A gender approach to three dimensions of governance—political-administrative governance, security governance, and socioeconomic governance—shows successes and problems in this post-conflict society. Special attention is given to the strategies of the women's movement in reaching gender-responsive governance. Some initiatives for new masculinities address the necessity of norm change in gender governance. The analysis of the literature and documents, supplemented by interviews, reveals the transformative potential of gender governance that solidly roots in women's rights and combines a multi-actor approach at grassroots, and at national and international levels with strong alliances and very concrete actions.

## Introduction

The challenging task of rebuilding the state starts functioning when war and armed conflict have ended, when armed groups have been dismantled, and when violence and destruction have stopped. Peace agreements define democracy hand in hand with new forms of governance. For women, huge opportunities arise in terms of inclusion in the new structures. However, reality shows that participation and representation of women remain deficient and that discrimination and violence against women continue in different manifestations than during the armed conflict/war.

After the wars on the territory of former Yugoslavia in the 1990's, the peace agreements or referendums created new states—Slovenia, Croatia, Serbia, the Former Republic of Macedonia/North Macedonia, Bosnia & Herzegovina, Kosovo, and Montenegro. In Bosnia & Herzegovina and Kosovo, international actors of the United Nations (UN), of the North Atlantic Treaty Organization (NATO), and of the European Union (EU) became responsible for security and state-building, whereas the other new states filled the political and administrative vacuum without much external assistance. Hereto, the Organization for Security and Cooperation in Europe (OSCE) assists all successor states with a comprehensive approach to security that encompasses politico-military, economic and environmental, and human aspects, including gender equality.

International organizations emphasized governance as a key instrument in building the new states and as a guarantee for sustained security (Ní Aoláin et al., 2011, p. 231). Good governance should serve economic development on the one hand, and avoid the recurrence of violent confrontations on the other. Linking development with security served also as the goal of gender governance, but feminist grassroot activists and femocrats in national and international institutions broadened this link with an emphasis on human rights. Notably, the Convention on the Elimination of All Forms of Discrimination against Women (CEDAW), the UN conferences of the 1990^1^ and the UN Security Council Resolution 1325 (UNSC, 2000) on WPS brought legitimacy to engender governance from the perspective of women's rights. In the post-conflict context that necessitates the creation or reform of the constitution and that requires the building of new government institutions governance with a focus on gender equality and women's rights, gender governance has the potential to improve the realities of women, but this potential requires a permanent struggle and is not smooth (Cornwall and Molyneux, 2006, p. 1176).

Focusing on the example of Kosovo, I show the dynamics, the successes, and failures around gender-responsive governance from the following: (1) from the perspective of human rights, and (2) from the perspective of UNSCR 1325 on WPS. By inserting gender into three dimensions of governance—political-administrative governance, security governance, and socioeconomic governance—I identify the actors, their alliances, and concrete activities that brought women's rights to public attention and expanded women's entitlements and capabilities. In addition, I claim that gender-responsive governance cannot avoid the confrontation with persistent patriarchal values and requires new interpretations of masculinity.

The purpose of this article is to show the importance of rights-based gender governance as it legitimates demands for justice, mobilizes for participation, rule of law, and accountability, and in the case of gender-based violence calls for prosecution and reparations.

In the following sections, I shall first present the conceptualization of gender-responsive governance with a focus on human/rights as the basic ingredient and shall proceed with a short presentation of two basic human rights-oriented instruments for post-conflict societies: CEDAW and the United Nations Security Council Resolution (UNSCR) 1325+ and the UN Security Council's resolutions regarding women, peace and security. Third, I'll turn to Kosovo and will show how gender equality advocates used those two human rights instruments for their claims of justice and security. Fourth, I will analyze with a gender lens three aspects of governance, that is, political-administrative, security, and economic governance. Last, in the conclusion, I shall turn to the issue of norm challenge and norm change toward new gender-equal masculinities.

### What Is Gender-Responsive Governance?

The concept of governance and of good governance was launched in the 1990's by the United Nations Development Programme (UNDP). UNDP defines governance as the exercise of economic, political, and administrative authority to manage the affair of a country at all levels. It comprises the mechanisms, processes, and institutions through which citizens and groups articulate their interests, exercise their legal rights, meet their obligations, and mediate differences (UNDP, 1997, p. 2).

First, situated within the notion of sustainable human development, the concept was soon broadly adopted for the Millennium Development Goals (2000–2015) emphasizing the “vital link between good governance, development, and human rights” (UNDP, 2014, p. 2). Good governance serves later also as a principle for the Sustainable Development Goals (2015–2030), adopted by all member states of the United Nations. “The new Agenda recognizes the need to build peaceful, just and inclusive societies that provide equal access to justice and that are based on respect for human rights (including the right to development), on effective rule of law and good governance at all levels and on transparent, effective and accountable institutions. Factors which give rise to violence, insecurity and injustice, such as inequality, corruption, poor governance and illicit financial and arms flows, are addressed in the Agenda” (United Nations, 2015, Section 35).

Governance relates to a “system of values, policies and institutions by which a society manages its economic, political, and social affairs through interactions within and among the state, civil society and private sector” (UNDP, 2014, p. 287). Mentioning the values of a society hints already toward the norm change embedded in the nine characteristics of good governance: participation, rule of law, transparency, responsiveness, consensus orientation, equity, effectiveness and efficiency, and accountability and strategic vision (UNDP, 1997). However, there is no uniform consensus about the importance of those characteristics, for example, “The UN, USAID, DFID, and the EC emphasize the social and political aspects of governance, highlighting the processes of participation, and responsiveness (to the needs of the people), democracy and human rights concerns. The banks and financial institutions focus more on economic governance, prioritizing transparency, accountability, and (public sector) efficiency and effectiveness” (Corner, 2005, p. 3). Although the indicators are gender-blind, several are of special importance to women's participation, rule of law, and accountability (Corner, 2005; UNDP, 2007, 19f).

After “governance” has found ground in the development discourse (World Bank, 2017), feminist writings were engaged with gender governance. Gender governance claims an inclusive process that includes the demands of women for rights and resources into policies and rules of implementation. In her overview report about gender and governance, Alyson Brody asserts that “(g)ender-sensitive governance requires that gender equality and the realization of women's rights are at the heart of the goals and practices of governance. Policies and legislation should address the differing needs, interests, priorities, and responsibilities of women and men, as well as their unequal economic and social power” (Brody, 2009, p. 2). Also, Niraja Gopal Jayal asserts in her essay that a gendered view of governance must be rights-based (Gopal Niraja, 2003, p. 102). This rights-based conception of governance obliges duty bearers to protect and guarantee rights, mandates governments to enable conditions for claiming rights, encourages to identify obstacles, and engages in process and outcomes (Gopal Niraja, 2003, 106f). With this focus on rights, engendered governance is rooted in formal state structures that can address also the informal structures of power in family and households (Gopal Niraja, 2003, p. 124). Women's rights would make governance effective, in the public as well as in the private realm (UNDP, 2012: 63).

Elaborating the practice of effective gender governance, UN Women, the United Nations Entity for Gender Equality and the Empowerment of Women (UNW), defines gender-responsive governance as “the management of public affairs in a manner that addresses the social relations that undermine women's capacity to participate in public decisions and responds to gender biases and patterns of exclusion” (UNW, 2012, p. 1). This response to gender biases would mean, according to philosopher Martha Nussbaum in her essay on governance, to emphasize the necessary change in social norms that undermine gender equality and women's empowerment (Nussbaum, 2003).

Gender governance claims an inclusive process that includes the demands of women for rights and resources into policies and rules of implementation. In her overview report about gender and governance, Alyson Brody asserts that “(g)ender-sensitive governance requires that gender equality and the realisation of women's rights are at the heart of the goals and practices of governance. Policies and legislation should address the differing needs, interests, priorities, and responsibilities of women and men, as well as their unequal economic and social power” (Brody, 2009, p. 2).

In the post-conflict context, governance concentrates on political-administrative governance, security governance, and socioeconomic governance, all connected to each other (Ní Aoláin et al., 2011, p. 233). The authors criticize that most post-conflict governance programmes concentrate on political-administrative governance, whereas security governance is reserved to programmes, such as Demobilization, Disarmament, and Reintegration (DDR), rule of law, and peacekeeping with little civil society participation. The third aspect, socioeconomic governance, is largely ignored except the promotion of economic liberalization (Ní Aoláin et al., 2011). International actors would see governance as an instrument to attain and maintain security, and in the long run, as conducive to development (Ní Aoláin et al., 2011, p. 231). Overall, as a sign of good governance, institutions should be “accountable, transparent, inclusive and responsive” to their citizens (Brody, 2009, p. 1). Gender-responsive governance reforms, therefore, focus on women's needs, livelihoods, rights, and participation as entry points and parameters for the political-administrative, security, and socioeconomic governance components discussed above (UNW, 2015b). In general, a gender-responsive approach to post-conflict governance would insert gender mainstreaming in all those three governance aspects.

The political-administrative domain of governance would focus in general on democratization and institution building: free elections, representative political parties, municipal and national level structures, such as Ombuds office, ministries, and specialized departments. Here, gender mainstreaming would mean equal opportunity for women and men to be elected, and the absence of family voting^2^. In order to guarantee women's representation in elections and public governance, usually a quota system is established. Although quotas for women receive skepticism or even outspoken resistance, without a quota for women rarely more than 25% of women can be found on electoral lists of political parties, in parliament and in administrative bodies, including the judiciary (Ní Aoláin et al., 2011, p. 239). The strongest instrument for gender mainstreaming in public governance is a Ministry for Women's Affairs or a specific department either attached to another ministry or directly in the prime minister's office or the chancellery.

Gender-responsive security governance would include women who were members of the armed forces or at least those affected by armed forces [Women Affected by Fighting Forces (WAFF)] in DDR programmes that also provide training and income generation. Rule of law agendas would draft or improve a legal framework in harmony with international conventions and would allow access to justice for women, reparations for victims of gender-based violence, and wartime rapes, and would promote to achieve inheritance and property rights, freedom from gender-based and domestic violence, and self-determination in matters of marriage, divorce, and reproductive rights (Ní Aoláin et al., 2011, p. 260). Furthermore, Security Sector Reform would be gender-sensitive—women police officers would need to be recruited, all police staff would receive training about women's rights. Specialists inside the police for human rights and domestic violence would need to be found, action plans with Standard Operating Procedures (SOP) to combat gender-based violence and human trafficking would need to be created, and for all this, a substantive legal basis would be required.

The third aspect of governance mentioned above, socioeconomic governance, follows mostly the thinking of economic liberalization as the assumed fastest way for poverty alleviation, economic growth, and reconstruction of the destroyed infrastructure. Feminist critique of the liberal economic agenda raises the gender-bias inherent in that approach, especially the neglect of unpaid care work (Elson, 1995; Bakker, 2014). However, a problematic macro-economic policy might include skills development and professional training, leading to income generation either through self-employment, small enterprise development, cooperatives and job creation in agriculture, industry, and services that are essential for women if they want to attain income and economic independence for sheer survival and a decent life.

An important non-governmental component for institution building is the creation of and the support to civil society, that is, the non-state actors referred to above. Civil society initiatives usually arise already during the armed conflict, either concentrating on survival, shelter, health, and education, or engage directly for peace and an end to violence. In many post-conflict societies, international actors support civil society initiatives for the belief in their contribution to democratization, their articulation of societal concerns, and as a corrective to former warring groups that demand access to and inclusion in governmental or administrative structures. Apart from faith-based groups, non-governmental organizations, the NGOs, grow out from civil society's work during the conflict. Big varieties of women NGOs in post-conflict societies can be observed; they engage for social, educational, economic, and political issues. They provide health, including reproductive health care, for women and also for victims of rape and domestic violence, and income-generation for women including war widows. They help the reintegration of displaced persons and former combatants engage in awareness-raising and advocacy for women's rights and in the fight against discrimination, and they campaign for reparations to war victims, and organize women's participation in post-conflict elections and monitor elections. Post-conflict NGOs and initiatives by women might have different visions, identity, and constituencies, and are not always free from tensions within and among them; nevertheless they are important actors in gender-oriented governance (OSCE, 2004; Kumar, 2010; Helms, 2013; Jacobson, 2013; UNW, 2015a).

That comprehensive gender governance agenda—political-administrative governance, security governance, and socioeconomic governance—envisions an ideal post-conflict transformation. The ideal lies in the belief that gender relations can rather smoothly be reformed because they have been shaken already during the armed conflict. Despite all the human pain, loss, and especially the experience of gender-based violence against women, armed conflict can bring also empowering effects for women due to their new multiple roles as civil and military actors (Lindsey, 2001, p. 31; Ní Aoláin et al., 2011, p. 41; Webster et al., 2019). Hope for gender equality rests, ironically, on the disruption of social, political, and economic life in situations of war and armed conflict and the assumption that after conflict, traditional gender relations will be newly negotiated on the basis of women's empowerment gained during the armed conflict and then fairly arranged.

Before I discuss below important elements of gender governance in Kosovo, I delineate two, in my opinion, basic ingredients of gender governance in post-conflict societies, CEDAW, and UNSCR 1325.

### Human Rights as Basis of Gender Governance

CEDAW is also called the “magna charta” of women's rights. Adopted by the UN General assembly in 1979, CEDAW defines discrimination against women and sets national agendas to end discrimination and encompasses all aspects of human rights. The Convention defines discrimination against women as “.any distinction, exclusion or restriction made on the basis of sex which has the effect or purpose of impairing or nullifying the recognition, enjoyment or exercise by women, irrespective of their marital status, on the basis of equality of men and women, of human rights and fundamental freedom in the political, economic, social, cultural, civil, or any other field”^3^.

The introductory text on the CEDAW website states that “(b)y accepting the Convention, States commit themselves to undertake a series of measures to end discrimination against women in all forms, including:

- to incorporate the principle of equality of men and women in their legal system, abolish all discriminatory laws, and adopt appropriate ones prohibiting discrimination against women;
- to establish tribunals and other public institutions to ensure the effective protection of women against discrimination; and
- to ensure elimination of all acts of discrimination against women by persons, organizations or enterprises” (Webster et al., 2019).

The gender governance elements of CEDAW lie in legal and institutional measures that must be presented to the CEDAW Committee. The mechanisms of CEDAW with regard to periodical, written, and direct reporting require government institutions, notably ministries, to inform whether women's needs and priorities have advanced. Through the participation of women's organizations in the reporting mechanism, gender-responsive governance becomes transparent, accountable, and inclusive, which are the characteristics of good gender-governance.

### UNSCR 1325+: UN Security Council Resolutions Regarding WPS

For conflict-related governance, the UN Security Council Resolutions regarding women, peace and security are the main instruments addressing times of war and peace alike.

In October 2000, the UNSC adopted Resolution 1325 on WPS. UNSCR 1325 is considered as a milestone for the protection of women against violence in the context of armed conflict/war and for the call to women's participation in peace negotiations and further inclusion in governance. The initial resolution, 1325, focuses on three main pillars: prevention of conflict and gender-based violence, protection against violence, and participation of women in peace, security, and conflict-related matters (3 Ps), thus emphasizing the human rights of women as related to bodily integrity and political inclusion. A fourth pillar refers to relief and recovery, specifically addressing the needs of women and girls in refugee camps and settlements.

Some specific paragraphs of UNSCR 1325 relate to our discussion of governance: UNSCR 1325 reaffirms the need to implement fully international humanitarian and human rights law that protects the rights of women and girls during and after conflicts; it (u)rges Member States to ensure increased representation of women at all decision-making levels in national, regional, and international institutions and mechanisms for the prevention, management, and resolution of conflict; and it (c)alls on all actors involved, when negotiating and implementing peace agreements, to adopt a gender perspective, including, inter alia: “(a) The special needs of women and girls during repatriation and resettlement and for rehabilitation, reintegration and post-conflict reconstruction,” (b) measures that support local women's peace initiatives and indigenous processes for conflict resolution, and that involve women in all of the implementation mechanisms of the peace agreements; “(c) measures that ensure the protection of and respect for human rights of women and girls, particularly as they relate to the constitution, the electoral system, the police and the judiciary” (UNSC, 2000, p. 3).

Thus, we see UNSCR 1325 calling for human rights of women during and after the conflict on legislative, judicative, and executive levels, women's decision-making at all levels as a matter of inclusion, and a gender perspective in all peace arrangements. Until today, the Security Council adopted nine additional WPS resolutions: 1820 (2009), 1888 (2009), 1889 (2010), 1960 (2011), 2106 (2013), 2122 (2013), 2242 (2015), 2467 (2019), and 2493 (2019). All those nine subsequent resolutions confirm UNSCR 1325 and expand to specific issues. Five of the resolutions (1820, 1888, 1960, 2106, and 2493) focus on sexual violence; six resolutions focus mainly on monitoring, expertise, and leadership (1888, 1889, 1960, 2122, 2242, and 2493). Monitoring, for which the UN and EU have developed indicators as demanded by UNSCR 1889, as well as expertise and leadership can also be seen as elements of governance. A strong monitoring instrument includes National Action Plans hitherto (August 2020)^4^ adopted by 86 Member States of the United Nations. Monitoring of the requirements of WPS is a task for all stakeholders—the United Nations, international organizations, national governments, and civil society organizations (McLeod, 2016). Again, transparency, accountability, and inclusiveness reflect the implementation of WPS governance prescriptions.

### Alignment of CEDAW and UNSCR 1325

In order to align CEDAW with UNSCR 1325, the CEDAW Committee adopted the General Recommendation No. 30 on women in conflict prevention, and in conflict and post-conflict situations in 2013^5^. This Recommendation gives guidance on “concrete measures to ensure women's human rights are protected before, during and after conflict. This General Recommendation makes clear that the Convention applies in all forms of conflict and post-conflict settings and addresses crucial issues facing women in these settings, including violence and challenges in access to justice and education, employment, and health”^6^. This means that states are required to include the WPS issues of UNSCR in their periodical reporting to the CEDAW Committee (UNW, 2015b).

The share of UNSCR on WPS with CEDAW, “the demand of women's participation in decision-making at all level, the rejection of violence against women as it impedes the advancement of women and maintains their subordinate status, equality of women and men under the law, protection of women and girls through the rule of law, the demand that security forces and systems protect women and girls from gender-based violence, the recognition of the fact that distinct experiences and burdens of women and girls come from systemic discrimination, and the demand to ensure that women's experiences, needs and perspectives are incorporated into the political, legal and social decisions that determine the achievement of just and lasting peace”^7^. Linking the WPS requirements with CEDAW and the General Recommendation 30 on women in conflict prevention, conflict and post-conflict situations reporting mechanism has increased the dialogue between the two regimes and “inter-regime accountability” (O'Rourke and Swaine, 2018, p. 199).

In review, although the WPS resolutions have received critique for focusing solely on women as victims and actors in peacebuilding, but ignoring intersectional differences among women and also for ignoring women as combatants, for glossing over hierarchical gender relations, for co-option into non-feminist power regimes and for not addressing militarism and masculinities (Puechguirbal, 2010; Holzner, 2011; Pratt and Richter-Devroe, 2011; SDC, 2013; McLeod, 2016), women peace activists embrace the WPS resolutions as a powerful instrument for acknowledging women's human rights and incorporating women in peace negotiations and in governmental functions which can be seen in the abundant publications collected by Women's International League for Peace and Freedom (WILPF)^8^.

### Gender Governance in Kosovo

In the following sections, First I provide a brief background of the history of the Kosovo conflict, the process to the country's independence in order to provide context and move then to the discussion about the implementation of CEDAW and UNSCR 1325. Mainly I use information from the government of Kosovo, the Kosova Women's Network (KWN)^9^, publications by the UN and OSCE, and my own interviews with key personnel.

The Yugoslav Constitution established the Autonomous Province of Kosovo and Metohija within the Yugoslav constituent republic of Serbia. Ethnic tensions between the minority Serb and the majority Albanian population had a long history that culminated in the late 1980's in the first popular uprisings and peaceful protests, but then also in the violent attacks of Albanian groups. The Milosevic regime of the Federal Republic of Yugoslavia ended the autonomy status of the province in 1990 and dismissed Albanians from all public functions. In response, the Albanian population founded underground schools and medical services, financed by the Albanian diaspora and the population. During that period, women played a strong role by providing food, education, and medical services. Going beyond providing services, women organized several huge demonstrations with up to 15,000 persons in 1998, protesting against Serbian blockades and violence (Farnsworth, 2008, p. 15).

During the 1990's, Kosovo Liberation Army (KLA), a rebel group was formed and resorted to armed struggle against Yugoslavian police stations and security personnel with smuggled weapons, which were looted from Albanian ammunition depots after the collapse of the so-called pyramid gambling scheme in 1997. Reports of violent retributions by the Serbian police and by paramilitary groups against the Albanian population caught international attention and response. In 1998, the OSCE entered Kosovo and attempted to reach a ceasefire, but failed. When reports of severe massacres became public in January 1999, NATO gave president Milosevic an ultimatum to withdraw his troops; after his refusal and the failure of negotiations, between March and June 1999, NATO forces bombed Serbian military and police structures in Kosovo and infrastructure in Serbian Yugoslavia, including Belgrade. Then the Serbian forces started a policy of ethnic cleansing, displacing hundreds of thousands of Albanians to neighboring countries. After 11 weeks of bombing, the Yugoslav regime agreed to withdraw from Kosovo, and on 10 June 1999, the so-called Kosovo Force (KFOR) consisting of NATO and several Non-NATO countries entered as peacekeeping force with 40,000 troops. Immediately, the country came under the control of the United Nations Mission to Kosovo (UNMIK), mandated by UNSCR 1244^10^. For assistance in state-building, many well-staffed organizations opened offices in Kosovo: the UNKT (United Nations Kosovo Team) with all its agencies and the two international financial institutions, the IMF and the World Bank, and many international NGOs like Caritas, CARE, and [Union Centrale des Coopératives Agricoles de l'Ouest (UCCAO)] a.o. ^11^.

UNMIK describes its mandate as “help the Security Council achieve an overall objective, namely, to ensure conditions for a peaceful and normal life for all inhabitants of Kosovo and advance regional stability in the western Balkans.” UNMIK has been divided into four sections which it calls “pillars.” These include the following:

- Pillar I: Police and justice (United Nations-led)
- Pillar II: Civil Administration (United Nations-led)
- Pillar III: Democratization and institution building (led by the Organization for Security and Co-operation in Europe)
- Pillar IV: Reconstruction and economic development (European Union-led).

KFOR became responsible for maintaining security and deployed thousands of military personnel in the country^12^.

Until 17 February 2008, when the Assembly of Kosovo declared unilateral independence, UNMIK was responsible for running the country. With that declaration of independence, supported by most EU member states^13^, the tasks of UNMIK were taken over by the EU, and the EU office in Kosovo, and a European Union Special Representative (EUSR). Building on the framework of the European Security and Defense Policy (ESDP), the European Union Rule of Law Mission in Kosovo (EULEX) was created. EULEX assists and supports the Kosovo authorities in the rule of law, specifically in the police, judiciary, and customs areas. In September 2012, international supervision ended, and Kosovo became responsible for its own governance, but UNMIK, KFOR, OSCE, and EULEX remain active in the country.

### CEDAW in Kosovo: Legal Basis for Gender Equality

Although Kosovo does not have the status of a UN member state, and thus cannot be obliged to report to the CEDAW Committee, it had adopted very early international obligations and laws directed at gender equality, non-discrimination, and protection of women against violence and abuse. A few months after the declaration of independence in 2008, the parliament of Kosovo ratified the Constitution of the Republic of Kosovo^14^. The text of the constitution is greatly based on the Constitutional Framework for Provisional Self-Government, signed by the Special Representative of the Secretary General of the United Nations in May 2001 (UNMIK, 2001).

The Constitution (Article 7) acknowledges gender equality as “a fundamental value for the democratic development of the society”. Moreover, Article 24 of the constitution maintains that all forms of discrimination are prohibited, including discrimination on the grounds of gender and sexual orientation as well. Article 22 of the Constitution about the Direct Applicability of International Agreements and Instruments says: “Human rights and fundamental freedoms guaranteed by the following international agreements and instruments are guaranteed by this Constitution, are directly applicable in the Republic of Kosovo and, in the case of conflict, have priority over provisions of laws and other acts of public institutions. Under this article, “The Convention on the Elimination of All Forms of Discrimination Against Women” is mentioned in a row with other international agreements and conventions [i.e., the European Convention on Human Rights (ECHR)]^15^.

The principle of incorporating equality between women and men is also mentioned in Article 24 of the constitution that guarantees “Equality before the Law^16^” in three paragraphs, with an explicit reference to gender in the second paragraph:

1. All are equal before the law. Everyone enjoys the right to equal legal protection without discrimination.
2. No one shall be discriminated against on grounds of race, color, gender, language, religion, political or other opinion, national or social origin, relation to any community, property, economic and social condition, sexual orientation, birth, disability, or other personal status.
3. Principles of equal legal protection shall not prevent the imposition of measures necessary to protect and advance the rights of individuals and groups who are in unequal positions. Such measures shall be applied only until the purposes for which they are imposed have been fulfilled.

Other constitutions that guarantee importance to women, include Article 37 and the Right to Marriage and Family^17^ that emphasizes equality of the spouses.

Already in 2004 (UNMIK, 2004), the Assembly of Kosovo adopted a Law on Gender Equality with the introductory text: “The present law shall preserve, treat and establish gender equality as a fundamental value for the democratic development of the Kosovo society, providing equal opportunities for both female and male participation in the political, economical, social, cultural and other fields of social life”^18^. In this law, a quota system was enshrined in Article 3.2 that states “Equal gender participation of both females and males, (...) is achieved in cases where the participation of the particular gender in the institutions, bodies or at the level of authority is 40%.” This law was lastly amended in 2015^19^, and the quota was extended to 50%, including all levels of decision-making “in political and public life” (Articles 3.1.15 and 6.8).

In addition, “gender responsive budgeting and gender mainstreaming in all institutional policies (for bodies “at all levels of legislative, executive, judicial and other public institutions)” are required (Morina and Farnsworth, 2015, n. p.). Other improvements lie in the definition of sexual harassment, stronger fines for violating the law, the obligations to collect gender-disaggregated data, clearer responsibilities of gender equality officers, fines for sexual harassment, broad definition of gender, (Morina and Farnsworth, 2015).

By this constitution, women of Kosovo enjoy *de iure* human rights in all aspects—political, economic, and social. For gender-responsive governance in Kosovo, the key standard of human rights as cited in UN Women's definition quoted above is achieved and further strengthened by the Law on Gender Equality. In addition, the Law on Protection against Domestic Violence, the Law on Prevention and Combating of Trafficking with Persons and Protection of Victims of Trafficking, the Law on Family and Social Services and the Criminal Code intend to protect victims of gender-based violence, to prosecute and punish offenders and to offer women various social services. Hence, the legal foundation for implementation was profoundly laid.

Since August 2019, also lesbian, gay, bisexual, transgender, and intersex people (LGBTI) rights are recognized when the Kosovo Court of Appeals officially approved the request of a trans citizen to change the name and gender marker in the Central Civil Status Register and Civil Status Principal Register^20^.

In the formulation of those laws and the action plans for implementation, women activists, notably the organizations united in KWN were involved, in line with its mission “to support, protect and promote the rights and interests of women and girls throughout Kosovo, regardless of their political and religious beliefs, age, level of education, sexual orientation, and abilities”^21^. EULEX and OSCE that have the mandate to give legal advice to the government of Kosovo assisted in the formulation of laws and related action plans.

To sum up, with the incorporation of CEDAW in the Constitution of Kosovo, the basis was laid for laws regarding gender equality and women's rights and their implementation by institutional mechanisms, action plans, and programmes.

### UNSCR 1325 in Kosovo

“We've used 1325 since 1999, even before such a Resolution existed” (Farnsworth, 2011, p. 5).

This quote reminds that Kosovo women continued their activism of the 1990's when Albanian citizens were dismissed from public functions, when the health care and school system broke down, and the country was tormented by the armed conflict. That activism persisted in the post-conflict period of the next decades until now.

After the war, women lobbied over and over again for participation in peace talks, referring to UNSCR 1325, but met much ignorance and refusal by international mediators (Villellas and Morena, 2008: 18). The publication, “1325 Facts and Fables” of the KWN^22^ illustrates vividly women's activism during those years (Farnsworth, 2011). Kosovo women activists had confronted the all male international negotiators for the status talks like Martti Ahtisari and Security Council members on their visit to Kosovo in 2007 with UNSCR 1325 and its enshrined call for women's participation. Letters sent to the officials were not responded; therefore, women chose to change the tactics and demonstrated on 8 March in the streets with the slogan, “No more flowers, we want power,” referring to the practice that in socialist countries on International Women's Day, women receive flowers from men (Farnsworth, 2011, p. 47). They used a banner saying “Resolution 1325 gives us the right to be in the negotiation team” (Farnsworth, 2011, p. 48). Even this effort was in vain; at the end, women were only permitted to join a few working groups. The final text of a new resolution following UNSCR 1244 that would guarantee Kosovo's independence included a reference to UNSCR 1325, but it “was not even put on the table of the Security Council because Russia said they would veto it” (Farnsworth, 2011, p. 51).

These frustrating experiences demonstrate on the one hand that Kosovo women consciously used UNSCR 1325 as a lobby and advocacy instrument, but that, on the other hand, influential and highly placed international negotiators were ignorant about this resolution and resisted acknowledgment of its content. That lack of respect to this instrument of the highest UN body, the Security Council, prevented the implementation of UNSCR 1325. This unawareness, unwillingness and resistance can only be explained by a “patriarchal” and “traditional” attitude of the male negotiators themselves, an attitude they accused of belonging to Kosovo society. Hence, uninformed, male-biased and orientalist perceptions of outsiders generated the exclusion of Kosovo women in talks about the future of the country.

Yet, the silence about UNSCR 1325 changed in 2010 at the occasion of the commemoration of 10 years of UNSCR 1325. Then, and in the following years, international organizations embraced the messages of the resolutions on WPS. Public events with stakeholders from UN, KFOR, OSCE, EULEX, EU, embassies, government and NGOs, notably KWN, transported the message of prevention, protection, participation, and recovery to their own staff and a wider public audience and promoted a.o. a zero tolerance of sexual violence^23^.

In 2014, the government of Kosovo under the guidance of the Agency on Gender Equality and with the participation of civil society, UN Women and Office of the High Commissioner of Human Rights (OHCHR) has adopted an Action Plan for the implementation of UNSCR 1325 for the period 2013–2015^24^ (Injac et al., 2014). The Action Plan integrates a call for women's rights and includes:

- Promote and implement Resolution 1325 in Kosovo, locally and regionally;
- Coordinate joint actions among institutions, civil society, and interest groups toward implementing Resolution 1325 and other programs that promote gender issues;
- Create a new premise for strengthening and promoting the rights that belong to women and girls in Kosovo;
- Integrate gender issues into the security sector;
- Create opportunities for promoting transitional rights in Kosovo;
- Exchange experiences with different countries in implementing the Resolution, contributing to women in peace and security issues;
- Enhance society's attention to and the commitment of institutions in mitigating the consequences of the war in Kosovo.

The Action Plan can be seen as a response to the critique of the KWN in their above-mentioned publication “1325 Facts and Fables” of 2011. That publication includes an assessment of the UN indicators on WPS with systematized, perception-based data. KWN developed scores from 0 (not implemented), 1 (partially implemented), 2 (fully implemented), and a category for “don't know.” Also, values for the progress or regress are given for the implementation of UNSCR 1325 during the decade 2001–2011.

The results of this assessment are slightly positive for an impressive range of obligations enshrined in the WPS resolutions: for direction issued by the heads of military and police in the peacekeeping missions and for the measures taken by Kosovo police to protect women's and girls' human rights, for more women in senior positions in UN and in other international field missions, by the representation of a negotiator (Ms. Edita Tahiri) for the EU-mediated Serb-Kosovo dialogue, for increased representation and meaningful participation of women in national and local governance, as citizens, elected officials and decision makers (though the legal quota has not been fulfilled), for the national laws on political, economic, social, and cultural rights in line with international standards, for operational mechanisms and structures to strengthen physical security and safety for women and girls, for the control of illicit small arms and weapons, and for trainings for security and justice personnel. Regarding indicators for relief and recovery, improvements were observed about the school enrolment of girls with the exception of the minority Roma, Ashkali, Egyptian, and Gorani girls, and access to reproductive health care has expanded. Transitional justice measures see progress, albeit insufficient, in the prosecution of crimes against women and witness protection (Subotic and Zaharijevic, 2018).

In KWN's evaluation study on UNSCR 1325, international organizations (UN, KFOR, OSCE, EU) also were asked to report about their gender mainstreaming activities. Some of those offices have gender advisors or at least gender focal points. Gender advisors in international organizations share the feeling of “marginalization and powerlessness” according to KWN's study, unless they have direct access to decision-makers (Farnsworth, 2011, p. 37). Finding allies among men in high positions as well as nominating men in the function of Gender Focal Point (Farnsworth, 2011, p. 37) compensate for the absence or low representation of women in those organizations. KFOR and also EULEX used this dual strategy for internal governance^25^.

Without a doubt, KWN was and is a driving force for the recognition of UNSCR 1325. The Network continuously organized activities, such as public debates, awareness campaigns, roundtables, media events, and the presentation of its publications for promoting Resolution 1325 in Kosovo and strengthened their demands by alliances with gender advisors in national and international organizations (KWN, 2014).

Despite these multiple efforts and the continuous lobby for the implementation of UNSCR 1325 and the WPS agenda also at international meetings of the EU and NATO^26^, the struggle for the implementation of UNSCR 1325 in Kosovo remained necessary. Recently, in autumn 2020, on the 20th anniversary of UNSCR 1325, KWN wrote a public letter to the EU Special Representative for Belgrade–Pristina dialogue with the demand to take part in the consultations with civil society in Kosovo concerning the dialogue between Kosovo and Serbia. KWN argues: “KWN and its members have been engaged in cross-border peacebuilding in the region, including Serbia, for 30 years. Our members include women's rights groups of all ethnicities. We monitor the situation and liaise with diverse women to identify their needs and priorities”^27^. Referring to the 20th anniversary of UNSCR 1325, the Network also addressed the own government to include women in the Dialogue with Serbia^28^. KWN criticized politicians at the national and EU level that they have not yet internalized their obligation to comply with national and international responsibilities of the WPS agenda. It is the strategy of the women's movement to steadily remind the power holders of their duties.

## Aspects of Gender Governance

### Political-Administrative Governance

The government of Kosovo has undertaken several political-administrative measures for gender governance. The independent institution of an Ombudsperson has been installed to hear complaints of human rights violations from individuals or entities in Kosovo. In 2004, along with the Law on Gender Equality, the Ombudsoffice established a specialized unit on gender equality. In the same year, the Office for Gender Equality (renamed later as Agency for Gender Equality, AGE) within the Office of the Prime Minister was created and each ministry appointed a Gender Equality Officer. At the level of local government, an Office of Gender Affairs with a Gender Affairs Officer in each municipality was installed^29^. In 2008, the Kosovo Program for Gender Equality (2008–2013), a strategic programme to institute gender equality in all policies and programs, including staffing, was approved. After many critiques about the lack of implementation of the Law on Gender Equality with regard to women's political participation called for a national programme and an alignment with the EU Gender Equality Acquis (Morina, 2017; Zymberi, 2017). The new, progressive government of Kosovo under Albin Kurti responded to the critique: In early 2020, he presented the final draft of the National Program on Gender Equality, together with the Amendment of the Legal Framework for Protection against Domestic Violence^30^.

An important official for gender equality was voiced by President Atifete Jahjaga, the first female president (2011–2016) in the region and a former deputy director of the Kosovo police. She spoke in public about gender equality and soon launched the establishment of a Center on Gender Studies and Research at the Faculty of Philosophy at the University of Pristina. She also founded the National Council for Survivors of Sexual Violence during the War in Kosovo. In her Foundation, established in 2018 after her period as president, she continued committing to women's empowerment and rights, social inclusiveness, interethnic and regional reconciliation, and security^31^.

In late 2012, President Atifete Jahjaga with the assistance of the US based National Democratic Institute (NDI) hosted the international conference on “Partnership For Change Empowering Women” that resulted in the Pristina Principles. The Principles listed a number of demands regarding gender equality in all sectors of society affirming the rights of women to political participation and representation, economic resources, women's property rights, and access to security and justice, and call for actions to make these principles a reality^32^ (BalkanInsight, 2012; NDI, 2012).

In assessing achievements and deficiencies, the Kosovo Country Gender Profile of 2014 draws a critical picture of political-administrative governance: Though the good legal framework for women's rights and inclusion, “(g)overnment institutions at all levels tend not to understand how to mainstream gender within their work. Gender equality officers within ministries and municipalities are marginalized; few are ever involved in programmatic planning, budgeting, impact assessments, and/or analyzing draft laws or policies from a gender perspective.” Other actors like women's civil society organizations are rarely consulted by international stakeholders active in Kosovo when setting priorities (Färnsveden et al., 2014, p. 7). The Kosovo Gender Analysis, conducted 4 years later, confirms the underrepresentation of women governmental functions and that “(m)ost primary and secondary legislation in Kosovo lacks a gender perspective, not targeting the potentially different needs and priorities of women, men, girls and boys” (KWN, 2018a, p. 1).

Voices of critique about the lack of implementation of the Law on Gender Equality with regard to women's political participation called for a national programme and an alignment with the EU Gender Equality Acquis (Morina, 2017; Zymberi, 2017). The new, progressive government of Kosovo under President Albin Kurti responded to the critique: In early 2020, he presented the final draft of the National Program on Gender Equality, together with the Amendment of the Legal Framework for Protection against Domestic Violence^33^.

These deficiencies in the implementation of the non-discrimination and equality laws extend also to the realm of justice: for the access of women to justice is hard especially with respect to inheritance, protection from domestic violence, and custody over children in case of divorce. Again, patriarchal attitudes are named to explain those deficiencies: “The effective enjoyment of women's rights is affected to some extent by patriarchal customs and tradition, but also perpetuated by weak rule of law” (Färnsveden et al., 2014, p. 19). These “patriarchal customs and traditions” originate in the *kanun*, a code of customary law, going back to the 15th century, which regulated rural societies in Albanian-speaking areas in the Balkans. The *kanun*, mildly said, is gender-biased: it assigns rights to the husband and duties to wives (Rexhaj, 2018, p. 35). Women's total submission to men in the patrilineal and patrilocal family arrangement allows domestic violence: “The husband has the duty to teach and yell at his wife, when she does mistakes, does not listen to him and when she does not do her belonging chores. When the wife shows contempt to her husband's words and when she is guilty and violent, then the husband has the right to punish her” (Rexhaj, 2018, p. 36). Due to the inability of the state to provide social security, the family economy, based on the patriarchal principle of assigning reproductive roles to women who also are held responsible for the honor of the family contributes to the perseverance of the patriarchal mindset in the *kanun* (Latifi, 2014). To conclude, political-administrative gender governance does not only require profound laws and government offices and officers, but also a bundled effort by state and non-state actors, critical researchers, and women's groups to install and implement national programmes with in-built accountability (UNIFEM, 2008). However, political-administrative gender governance faces the problem of legal pluralism, when legal norms of the state are undermined by traditional social norms.

### Security Governance

Gender-responsive security governance in Kosovo has advanced very far: Security Sector Reform increased the number of police women (in March 2016 at 14%^34^) who have formed an association with 600 members for capacity building, outreach, networking, and partnerships (Stickings, 2015). A special human rights unit and a special domestic violence unit have been installed in the police, and laws and action plans with SOPs are drafted. A National Coordinator ensures cooperation and coordination among institutions to combat domestic violence and human trafficking, respectively (Färnsveden et al., 2014). In the Ministry of Justice, a Victims Protection Division is established. Free Legal Aid offices exist in several regions. Nine partly government, partly donor funded shelters for battered women and children are established countrywide; thus an anti-domestic violence structure is in place. In addition, national strategies for ensuring security governance for women complement the gender equality and the anti-discrimination laws released, include National Strategies against Trafficking in Human Beings from 2011 onwards^35^, a National Strategy and Action Plan against Domestic Violence also from 2011 onwards, and the already mentioned Action Plan for the Implementation of UNSCR 1325.

In the following section, I discuss the two most salient features of women's bodily security–domestic violence and wartime sexual violence.

#### Domestic Violence

With regard to women's personal security, high incidences of domestic violence appear in public debates with big concern (Government of Kosovo, 2016). Already in 2008, KWN published the study “Security begins at home. Research to inform the national strategy and action plan against domestic violence in Kosovo” (KWN, 2008). In 2015, KWN published a similar study titled “No more excuses. An Analysis of Attitudes, Incidence, and Institutional Responses to Domestic Violence in Kosovo”. Both studies “assess citizen awareness of domestic violence, incidence, and institutional response” (KWN, 2015, p. 5).

According to the 2008 study, ~43% women and men of the respondents said that they had suffered domestic violence in their lifetimes, but the figure in 2015 is much higher: 62% in total, 68% women, and 56% of men. On average, annually 1,000 women report domestic violence to the police (Färnsveden et al., 2014, p. 46). Economic dependence by women on male household heads and prevalence of women's reproductive role in gender relations are seen as the main reason of domestic violence. Girls as well as boys are disciplined often violently into moralizing gender roles as mothers, respectively as bread-winners in line with the *kanun* (UNICEF Kosovo, 2014: 70; Qosaj-Mustafa, 2015, p. 10). Whereas, women victims suffered mainly violence from their spouses, male victims suffer from their fathers. Both instances can be attributed to the role of the autocratic father. Also, a more recent survey on men's attitude toward gender equality revealed that 12% of men have exerted violent behavior toward their female partners and one of 20 have admitted engaging in unconsented sexual relations (UNFPA/OSCE, 2018, p. 5).

Patriarchy does not only mean “rule of the father” but refers also to a social ideology (Lerner, 1986). Many women have internalized men's “right” to hurt them as the Demographic, Social, and Reproductive Health Survey of Kosovo^36^ has revealed: in case of leaving the house without the permission of the husband, neglect of children, quarrels, refusing sex, and even for burning food, female respondents find beating/striking acceptable. Although the study shows differences in terms of urban/rural residence, education, age, and ethnicity, in comparison, surprisingly, more women approve domestic violence due to their own “misbehavior” than men, ranging from 5 to 30% of the respondents, depending on their age: approval is less among the younger population. The lesser approval by men might be due to the effect of social desirability in answering; however, the data show an appalling attitude toward domestic violence (Statistical Office of Kosovo, 2009, p. 80). Also, the 2015 KWN study found that 21% of Kosovo citizens (men and women) agree that “sometimes it is okay for a husband to hit his wife” (KWN, 2015, p. 5).

Problems with regard to mitigating domestic violence include, “underreporting of violence by citizens; inadequate services available for persons including weak rehabilitation and reintegration programs; insufficient sustainability of shelters; poor enforcement of measures, such as child alimony; some inadequately trained or poorly performing persons within institutions; insufficient human and financial resources in some institutions; a lack of professional psychologists; inadequate coordination among institutions in domestic violence case management; and traditional gender norms that contribute to “blaming the victim” and provide an enabling environment for violence to continue” (KWN, 2015, p. 5). Some of these problems are related to human resources, some to insufficient structures, and some to misogynist attitudes (Council of Europe, 2017). In addition, women face difficulties to access the labor market due to few employment opportunities and often have to return home after their stay in a shelter for lack of other perspectives (Färnsveden et al., 2014, p. 23).

As a response to the widespread domestic violence, the Kosovo Agency for Gender Equality together with other government agencies in cooperation with civil society and academia evaluated the National Action Plan against Domestic Violence for the years 2011–2014 (Government of Kosovo, 2015). This Action Plan “addressed the needs of victims of domestic violence by instructing the response of Kosovo institutions on three main pillars: (Government of Kosovo, 2015) (1) Prevention, (2) Protection and Security, and (3) Support, Treatment and Reintegration” (Qosaj-Mustafa, 2015, p. 11). The National Coordinator against Domestic Violence, supported by UN Women, produces annually a list that marks the challenges concerning the understanding and implementing the legal framework and the functioning of the institutional mechanisms (Qosaj-Mustafa, 2015, p. 16). Consultation with civil society, shelter staff, legal experts, and international representatives resulted in an updated Strategy and Action Plan on Protection from Domestic Violence for the years 2016–2020^37^ with detailed objectives and indicators. Furthermore, the new Criminal Code, dated 14 April 2019, provides that violent offenses when committed within the family relationship are considered aggravating circumstances and punished with higher sentences (AGE, 2019, p. 121).

In order to improve practice, the Kosovo Agency for Gender Equality published in 2019 an “Assessment of the level of implementation of the Standard Operating Procedures for Protection against Domestic Violence in Kosovo”^38^. It recommends, in line with the Istanbul Convention against violence against women and domestic violence^39^, training for prosecutors and judges regarding the Standard Operating Procedures (SOPs) in order to raise their efficacy in dealing with such cases. Also, the police should have at each station a special interviewing room for victims of domestic violence, and protection orders should be routinely monitored.

On the initiative of UN Women, an interagency working group for gender equality and security with NGO representation and inclusion of the government's Agency for Gender Equality and the police officer in charge for cases of domestic violence was created in 2010 and persisted since then. The working group exchanges information on initiatives and cooperates in eventually joint activities like International Women's Day and the “16 Days of activism against gender-violence/violence against women.” The working group can be seen as an informal governance activity of state and non-state actors, expanded by members of international organizations, who all adhere to inclusive and responsive governance principles.

In conclusion, feminist women broke the topic of domestic violence out from the sphere of taboo first through conducting research to manifest the magnitude of the problem, then informing the public, addressing policy-makers and governmental institutions, and lobbying them for concrete steps of improvements, which finally the government siphoned into laws, strategy, and action plans that became instruments of accountability. We see that gender governance in Kosovo starts with a push from civil society before the implementation of Act from governmental institutions. Demands call for the improvement of the legal framework, policies and procedures, awareness of officials, and the society as a whole, institutional and public support for victims/survivors^40^.

#### Wartime Rapes

Security governance in post-conflict societies addresses also the prosecution of gender-based violence during war. In Kosovo, number of wartime rapes are estimated between 10 and 45,000 (HRW, 2000; Smith, 2000; Kosova Women's Network, 2011, p. 82; OHCHR, 2013, p. 40), but only a few prosecutions and even less convictions were effected so far (BalkanInsight, 2012; AI, 2017, p. 7; Haxhiaj and Martinovic, 2020).

In 2012, KWN started a campaign for justice to victims of war rapes on the occasion of an 8 March celebration organized by EULEX, who had at that time the mandate to prosecute war criminals. KWN handed over a letter with recommendations to the present EULEX Head of Mission for fast and sensible prosecution of suspects and comprehensive victim/witness protection. He had distributed roses to the women attending the celebration, but was with this letter persuaded to utter a supportive statement^41^. Also, the Amnesty International pressured EULEX to prosecute wartime rape (AI, 2012). Prosecuting wartime rape is very difficult as there are no DNA traces, and prosecutors have to rely on statements by victims and witnesses. Both, victims and witnesses and especially victim-witnesses (those victims who are called to court to give testimony what happened to them) usually are traumatized what might lead to inaccurate memories, or they are reluctant to report due to feelings shame, guilt, or timidity. Such limitations may be used by the defense to question the reliability of victims/witnesses with the consequence that the case is dismissed for lack of evidence^42^.

Crucial governance actors, the then Minister for Integration, Vlora Citaku, and especially the president of Kosovo, Atifete Jahjaga, who has founded For Survivors of Sexual Violence during the War in Kosovo, supported the call for justice for victims of sexual violence during the war. The president had used diligently her position by receiving victims in her office, reminding the police and the judiciary of their responsibility, and addressing the public with the message that the victims deserve support and care (CSIS, 2019).

With the highest governmental support and intense lobbying by women's groups and UN Women, in March 2014, the “Law on the Status and the Rights of the Martyrs, Invalids, Veterans, Members of the Kosovo Liberation Army, Civilian Victims of War and their Families” included also women survivors of sexual violence as civilian survivors of war. This law is important as it opened the door for reparations. Several ways to find a modus for reparations without stigmatizing or endangering wartime rape victims can be found in the consultancy report^43^ commissioned by OHCHR Kosovo (OHCHR, 2013). A Verification Commission, which involved two NGOs (Medica Kosova and the Center for Victims of Torture), who had helped the survivors for many years with counseling and material relief and established application criteria for the victims of war-time sexual violence, who are legally entitled to a monthly pension of € 230,- (AI, 2017, p. 35). Yet, access to justice for survivors and poor judicial performance in the prosecution of those war crime suspects are still matters of concern, because the stigma adhered to sexual violence prevents many women to come forward (AI, 2017, p. 6). Amnesty International also criticizes that the limitation of the period for eligibility for the period between 27 February 1998 and 20 June 1999, the official period of the war “excludes most Kosovo Serb women and girls, and Roma, Ashkali and Egyptian women and girls, who were predominantly raped after the end of the war, as well as some Kosovo Albanian women, perceived to have been disloyal to the KLA” (AI, 2017, p. 44).

In order to increase the compassion for victims of sexual violence during the war, Medica Kosovo, the NGO which assisted war victims of sexual violence already since 1999, had invited an artist to assist victims painting their pain on canvas as a form of art therapy. An exhibition of those paintings was shown in Kosovo and has also been brought to London at the “Global Summit to End Sexual Violence in Conflict” in June 2014 in the presence of the Kosovo President Atifete Jahjaga. Some months later, in 2015, artist Alketa Xhafa-Mripa organized a public art installation, called *Thinking of You*, in the soccer stadium in Pristina, where 5,000 skirts and dresses were hung on 45 clothes lines. Women from the whole country, including the president A. Jahaga had donated the clothes. “The laundry is washed clean, like the women who are clean and pure—they carry no stain,” she said (Cole, 2015). Yet, despite the message of the art installation that wartime rape should not be a taboo and that raped women deserve compassion and justice, the installation also serves as a nationalist project that reproduces gender asymmetries: the soccer stadium as a masculine space and feminine clothing representing women's sacrifice for the sake of the Kosovo-Albanian nation (Krasniqi et al., 2020).

In the same year, 2015, in the city center of the capital, a tall statue, named, Heroinat (heroine) was erected, portraying a woman's face. The statue should highlight women's sacrifices during the war and thus appropriates the masculine notion of hero toward women as victims but also as survivors. It is made of 20,000 pins, each pin a miniscule portrait of the monument, and referring to the alleged number of raped women during the war (Ferizaj, 2015a; Krasniqi, 2020).

So far, critique of the slow judicial process has continued: at a hearing at the US congress in 2019, a survivor of war rape together with the former president, Atifete Jahjaga criticized the failure of the judicial system to prosecute sexual violence carried out during the armed conflict (Travers, 2019). Criticizers refer also to the persistence of the *kanun*, the 500 years old traditional, male-oriented customary law that regulated social and family life through a strong emphasis on family honor. The public awareness actions of the paintings, the laundry exhibition and the Heroinat statue confront the old social norms of women's submission under the male rule.

These examples show that gender-oriented security governance in Kosovo needed strong pressure from civil society, foremost by women's organizations. Awareness creation, advocacy, and lobbying, high-level political support, especially by women of power, alliance building with government institutions and with international organizations made public awareness, empathy, and prosecution of some suspects/war criminals possible. Gender-aware security governance with all these components entails also “emotional governance” by reaching out to the souls of the population for compassion with victims.

### Socioeconomic Governance

Kosovo is one of the poorest countries in Europe: based on the data of the Household Budget Survey 2017, it is estimated that 18.0% of Kosovo's population lives below the poverty line, with 5.1% of the population below the extreme poverty line, living on €1.85 and €1.31, respectively per day (KAS, 2019). Hence, one quarter of the population is considered poor. Women are especially deprived in terms of access to employment, resources, and power and voice. The employment rate is at 12%; only 8% of property owners are women and 6% of the positions with decision-making authority in the public sector are held by women (Embassy of Sweden, 2017, p. 7). Women's rights to property and inheritance are continuously violated (OHCHR, 2020: 22). Apart from the generally restricted labor market, women experience many barriers for work outside the home: their traditional role as the primary caregiver in the family, lack of care services for the children, lack of flexible working arrangements, and favoring of male candidates over female candidates for private sector jobs (UNDP, 2016; KWN, 2018b). To combat some of those barriers, KWN supports with small grants women's economic initiatives (KWN, 2018b).

This bleak economic situation of women, coupled with low levels of education and inadequate skills for employment bring women into a situation of economic insecurity and into dependence on family resources. Thus, it is understandable that battered women return to violent husbands for lack of any other choice of sustenance (Krasniqi, 2014). Mitigation of economic dependence would require better education and well-designed employment schemes for women. Essential are skills development and professional training, leading to income generation either through self-employment, small enterprise development, cooperatives, and job creation in agriculture, industry, and services (World Bank Group in Kosovo, 2015).

Such approaches can, for instance, be found in the Kosovo's European Reform Agenda (ERA) from 2016 that intends to increase the labor market participation of women. Similarly, the sector strategy 2018–2022 of the Ministry of Labor and Social Welfare aims at reducing the level of unemployment and economic non-activity, in particular, focus on women, in addition to young people and other marginalized groups (World Bank, 2018). Nation-wide, government-directed initiatives demonstrate public awareness of women's under-representation in the labor market along with the necessity to strengthen women's productive and professional role, aside from their reproductive responsibilities in household and family.

In addition to state-led socio-economic governance schemes, there are some impressive income-generating initiatives by women. Two of them I present below.

In the village of Kruje, where a big massacre took place during which most of the men were killed and all buildings burnt, one of the widows, Fahrije Hoti, founded already in 2002, the Association of Women Farmers named, “Krusha e Vogel.” One-eighty women from the Association produce *ajvar* (a relish of red peppers and aubergine, very popular in the Balkans) and a variety of pickles from vegetables and fruits; they raise cattle and sell the milk (KWN, 2016). The founder declares in an interview that she always wanted to work and did not want to depend on charity, but that she had to overcome many prejudices about widows. “But I triumphed and had great success!” (OSCE and Ministry of Foreign Affairs of Finland, 2015; video).

Up in the southern hills of Dragash, the midwife Gjejrane Lokaj established the interethnic Albanian and Gorani-Bosnian Women's Initiative Association. Women and adolescent girls are brought together for training in tailoring, health care, and women's rights, especially about the laws on marriage, education, and inheritance. The activism of the women reached out to village politics when the group presented a whole list of demands to the male candidates for the election of the mayor with the announcement that if the then elected mayor would break his promises, the women would make sure that he would not be elected again^44^.

These two examples show that economic security must not only rely on political-administrative security, but even more so on overcoming the gender ideologies around women's “place,” virtue and honor, hence the dominant social gender norms (Nussbaum, 2003). Individual initiatives can and do break the barriers of those norms, which are entrenched in limited economic opportunities.

## Conclusion

In the discussion of the three dimensions of gender governance—political-administrative governance, security governance, and socioeconomic governance—we see that in the political-administrative sphere, the cooperation between state and non-state actors created a solid legal basis for gender equality with adherent implementation structures. Women activists called on those structures when addressing the two aspects of gender-oriented security governance discussed here, i.e., combating domestic violence and the struggle for justice for wartime rape survivors. Their actions targeted the prevalent social norms of family honor, the lack of compassion for victims, and the right to reparations. Also for gender-focused socioeconomic governance, women break the social norms that relegate them to the private sphere. The examples show that women succeeded in entering the labor market and in generating income either through government-supported schemes or through private initiatives.

Ní Aoláin et al. (2011, p. 253) stated that in the post-conflict period, “the strongest emphasis must be placed upon protecting women's rights, the post-conflict work in a human rights framework, fostering equality and preventing future discrimination.” In Kosovo's post-conflict era, gender governance rests solidly on women's rights: CEDAW as a legal basis and UNSCR 1325 as a tool are supplemented with administrative structures, national strategies, programmes, and action plans in order to guarantee a gender inclusive approach. But, it seems as if gender governance in Kosovo would float on a pendulum of success and failure. Success lies in the creation of a comprehensive, action-oriented legal system, in a very vocal women's movement, and in a supportive alliance between international and national actors, and between state and non-state actors. Failure lies, as so often, in insufficient implementation of the laws and weak accountability mechanisms to which the women's movement acts as an opponent (Pierson and Thomson, 2018). This opposition is then challenged with the creation of those strong alliances.

In order to make the government accountable, the Kosovo women's movement chooses for continuous public advocacy and smart lobbying in addressing “gender biases and patterns of exclusion,” seen as so central for gender-responsive governance (UNW, 2012, p. 1, see page 2). This started soon after the war when the new constitution was drafted, and when international stakeholders were reminded of their responsibility to follow UNSCR 1325. Consequently, the women's movement persistently reminded the various governments to take gender equality seriously. Advocacy and lobbying continued with campaigns to end domestic violence and to prosecute wartime rapes. Women's NGOs, also those founded for victims of the war, entered the business sector and created some employment and income opportunities for women. The strong KWN and its 153 member organizations, its charismatic director, Igballe Rugova^45^, its representative board, which includes also a few men, and its diligent approach to governance through documentation, advocacy, and lobbying is the loud voice for gender-responsive democracy and justice. Lately, also artists entered the scene of gender governance to reach the awareness and conscience of the society not only symbolically expressed women's grievances but also women's solidarity was exposed in paintings and installations. We can conclude that in Kosovo, the transformative power of gender governance combined a multi-actor approach at national and international levels, strong alliances, commitment on the highest political level, and very concrete actions.

For the problems, deficiencies, and failures in gender governance in post-conflict societies, gender relations in a patriarchal gender order are held responsible, especially the social norms that undermine gender equality and women's empowerment (Nussbaum, 2003). From the experiences in Kosovo, we can ask many questions. Why are patriarchal gender relations so sticky? Why is the gender habitus (Bourdieu, 1998) of masculine domination so pervasive? Is it due to the adherence to the gender norms stipulated in the *kanun*, the old customary law, is it male bonding practices, is it the loyalty among former male combatants, is it the gender division of labor, is it hierarchal family ties with a patriarch as the highest decision maker, is it the internalization of a male-dominated gender order in both women and men? The complexity of the gender habitus in Kosovo generates multiple questions each of which deserves elaborate scrutiny. For this scrutiny, attention to masculinities is a valuable avenue.

Ní Aoláin et al. (2011, p. 253) called for the identification of masculinities in the post-conflict context “in order to reveal and yield actual opportunities for women to gain agency and traction within the political and economic sectors.” The identification of the so-called “toxic” masculinities has been addressed already successfully by the “Young Men Initiative” in Kosovo. Schoolboys of the age 13–19 are joining clubs where they reflect in workshops about sexuality, non-violence, gender roles, non-discriminative behavior toward girls and women, and caring behavior, and engage in social activities (CARE, 2013; Bates, 2014). A similar project, called “Be a Man,” uses also peer education to challenge misogyny rooted in the *kanun* (Ferizaj, 2015b). Breaking gender stereotypes is also attempted by the initiative “Men in the Kitchen” (KWN, 2021).

Such initiatives can shatter to a certain degree the “stickiness” of the patriarchal order as testimonies of participants in those initiatives reveal. Nevertheless, as said above, governance for gender equality requires a multi-actor approach with strong alliances and persistence in identifying and addressing cases of gender discrimination in the political-administrative, security, and socioeconomic spheres.

## Author Contributions

The author confirms being the sole contributor of this work and has approved it for publication.

## Conflict of Interest

The author declares that the research was conducted in the absence of any commercial or financial relationships that could be construed as a potential conflict of interest.

## Footnotes

^1^World Conference on Human Rights 1993, Vienna; United Nations Conference on Population and Development 1994, Cairo; Fourth world Conference on Women 1995, Beijing.

^2^Definition of family voting: “a family member casts votes on behalf of the entire family or where one member of the family pressures other members to vote for a certain candidate” (iKNOW Politics, 2019 no date, p. 1).

^3^CEDAW, Available online at: https://www.un.org/womenwatch/daw/cedaw/cedaw.htm, p. 1.

^4^https://www.peacewomen.org/member-states

^5^https://www.unwomen.org/-/media/headquarters/attachments/sections/library/publications/2015/guidebook-cedaw-general-recommendation-30-women-peace-security-en.pdf?la=en&vs=1850 (accessed April 23, 2020).

^6^https://www.unwomen.org/en/digital-library/publications/2015/8/guidebook-cedawgeneralrecommendation30-womenpeacesecurity, p.1

^7^https://web.archive.org/web/20120501140626/http://www.gnwp.org/wp-content/uploads/2010/11/Cedaw-1325-1820-synergy2.pdf (text slightly adapted by the author).

^8^https://www.peacewomen.org/who-implements

^9^I use here the official designation Kosova by KWN, which is the Albanian name; otherwise I use the international official name for the country: Kosovo that does not prioritize ethnicities.

^10^For more details, see Available online at: http://www.britannica.com/event/Kosovo-conflict and https://en.wikipedia.org/wiki/Kosovo_War

^11^FAO, ILO, IOM, OHCHR, UNWOMEN (formerly UNIFEM), UNCTAD, UNDP, UNDSS, UNEP, UNESCO, UNFPA, UN-HABITAT, UNHCR, UNICEF, UNIDO, UNODC, UNOPS, UNV, WHO.

^12^Until today, there are still 3,500 KFOR troops in Kosovo. See Available online at: https://en.wikipedia.org/wiki/Kosovo_Force

^13^Until today, five EU member states have not acknowledged Kosovo's independence; these countries include Cyprus, Greece, Romania, Slovakia, and Spain. As of 4 September 2020, 116 UN member states have diplomatically recognized Kosovo, of which 15 have withdrawn their recognition. See Available online at: https://en.wikipedia.org/wiki/International_recognition_of_Kosovo

^14^https://en.wikipedia.org/wiki/Constitution_of_Kosovo

^15^http://www.gjk-ks.org/repository/docs/Kushtetuta_RK_ang.pdf, p. 6.

^16^http://www.gjk-ks.org/repository/docs/Kushtetuta_RK_ang.pdf, p. 7.

^17^http://www.gjk-ks.org/repository/docs/Kushtetuta_RK_ang.pdf, p. 11.

^18^http://www.kuvendikosoves.org/?cid=2,191,103

^19^https://gzk.rks-gov.net/ActDetail.aspx?ActID=10923, and https://equineteurope.org/wp-content/uploads/2019/10/Annex-LAW_NO._05_L-020_ON_GENDER_EQUALITY.pdf

^20^https://www.lgbti-era.org/news/kosovo-court-appeal-takes-historic-decision-legal-gender-recognition

^21^Quote taken from https://womensnetwork.org/programs/

^22^Kosova is the Albanian word for the country. In official texts, Kosovo is used.

^23^See for example the press release of EULEX in October 2010 under http://www.eulex-kosovo.eu/en/pressreleases/0101.php

^24^https://www.peacewomen.org/sites/default/files/kosovo_nap_2014.pdf (in Albanian, Serbian, English).

^25^Personal observation of the author.

^26^https://womensnetwork.org/?s=1325

^27^https://womensnetwork.org/resolution-1325-requires-consultations-with-womens-rights-groups-in-kosovo/ (accessed October 31, 2020).

^28^https://womensnetwork.org/resolution-1325-requires-consultations-with-womens-rights-groups-in-kosovo/

^29^http://www.stopvaw.org/

^30^https://womensnetwork.org/the-agency-for-gender-equality-and-government-pledge-to-advance-gender-equality/Kurti's presidency lasted (accessed 3 February 2020 until 3 June 2020)

^31^https://www.facebook.com/JahjagaFoundation/posts/162560131223409

^32^https://www.ndi.org/kosovo-summit-pristina-principles

^33^https://womensnetwork.org/the-agency-for-gender-equality-and-government-pledge-to-advance-gender-equality/ Kurti had to step down in spring 2020, a new government was formed but due to the arrest of the President Hashim Thaci in November 2020 and his indictment by the Kosovo Special Chambers in The Hague for alleged war crimes; this government fell and new election is foreseen in February 2021.

^34^https://www.eulex-kosovo.eu/?2,26,398

^35^National Strategy against Trafficking in Human Beings 2011–2014, Available online at: https://www.legislationline.org/download/id/5025/file/Kosovo_National_strategy_action_plan_against_THB_2011-2014_en.pdf, and National Strategy against Trafficking in Human Beings 2015–2019, http://www.kryeministri-ks.net/repository/docs/NATIONAL_STRATEGY_AGAINST_TRAFFICKING_IN_HUMAN_BEINGS_IN_KOSOVO.pdf

^36^https://ask.rks-gov.net/media/1832/demographic-social-and-reproductive-health-survey-in-kosovo-2009.pdf, p.78 f.

^37^https://md.rks-gov.net/desk/inc/media/52BA49FC-80C2-4172-A2F7-9E83D078F3E7.pdf

^38^https://abgj.rks-gov.net/assets/cms/uploads/files/Raporti%20ABGJ%20-%20Versimi%20i%20nivelit(1).pdf

^39^https://www.coe.int/fr/web/conventions/full-list/-/conventions/rms/090000168008482e

^40^https://womensnetwork.org/programs/gender-based-violence/

^41^Personal observation of the author.

^42^Personal communication of the author with the Head of Kosovo's Special Prosecution Office, August 2012.

^43^Title: *Healing the Spirit: Reparations for Survivors of Sexual Violence related to the Armed Conflict in Kosovo*.

^44^Personal communication of the author with Gjejrane Lokaj.

^45^See her portrait in https://www.eulex-kosovo.eu/?page=2,26,806
